# Serum Alanine Aminotransferase Levels within Normal Range Have Different Associations with Augmentation Index and Other Cardiometabolic Risk Factors in Nondrinkers and Drinkers: A Chinese Community-Based Analysis

**DOI:** 10.1155/2017/3689827

**Published:** 2017-05-09

**Authors:** Shihui Fu, Chunling Liu, Leiming Luo, Ping Ye

**Affiliations:** ^1^Department of Geriatric Cardiology, Chinese People's Liberation Army General Hospital, Beijing 100853, China; ^2^Department of Cardiology and Hainan Branch, Chinese People's Liberation Army General Hospital, Beijing 100853, China; ^3^Department of Nephrology and Hainan Branch, Chinese People's Liberation Army General Hospital, Beijing 100853, China

## Abstract

**Background:**

To investigate whether serum alanine aminotransferase (ALT) levels within normal range were associated with augmentation index (AIx) and cardiometabolic risk factors in nondrinkers and drinkers in Chinese community-dwelling population.

**Methods:**

There were 4165 participants with serum ALT levels within normal range.

**Results:**

Alcohol drinking was observed in 1173 participants (28.2%). In multivariate analysis, serum ALT levels of nondrinkers were independently associated with age, sex, body mass index (BMI), hypertension, diabetes mellitus, diastolic blood pressure, triglyceride, low-density lipoprotein-cholesterol (LDL-c), and AIx, while serum ALT levels of drinkers were independently associated with age, sex, BMI, triglyceride, and LDL-c (*p* < 0.05 for all).

**Conclusions:**

Associations of serum ALT levels within normal range with age, sex, body height and weight, and blood lipid were simultaneously present in participants with and without alcohol drinking, while associations of serum ALT levels within normal range with AIx, blood pressure, and glucose were seen in nondrinkers rather than in drinkers. These findings not only provide the evidence that serum ALT levels, even within the normal range, have different associations with arteriosclerosis and cardiometabolic risk factors in nondrinkers and drinkers but also are helpful in understanding the underlying pathophysiologic mechanisms linking the hepatic function to arteriosclerosis and cardiometabolic risk factors.

## 1. Introduction

Concerns about cardiometabolic risk factors have become a focus of public health researchers and practitioners. An elevation in serum alanine aminotransferase (ALT) levels has been suggested to signify the presence of abnormal hepatic function in patients with cardiometabolic risk factors [1]. Serum ALT levels have also been reported to be associated with cardiometabolic risk factors, including age, sex, body height and weight, and blood pressure, glucose, and lipid [2–5]. But these associations are not full analyzed within normal range of serum ALT levels, and thus, the significance of serum ALT levels within normal range needs to be evaluated in relation to these cardiometabolic risk factors [6]. Moreover, the studies in clinical patients cannot reflect the associations between serum ALT levels and cardiometabolic risk factors in community-dwelling population, and these associations may differ by ethnicity [7–9]. However, few large-scale studies focusing on these associations have been performed in Chinese community-dwelling population.

In addition to traditional cardiometabolic risk factors, augmentation index (AIx) is a significant indicator of arterial compliance, and a decline in AIx is paralleled with increased arteriosclerosis [10–12]. However, to our knowledge, there is no study evaluating the association between serum ALT levels and AIx all around the world. Meanwhile, the associations of serum ALT levels with AIx and cardiometabolic risk factors have been affected by alcohol drinking. Accordingly, the present analysis investigated whether serum ALT levels within normal range were associated with AIx and cardiometabolic risk factors in nondrinkers and drinkers in Chinese community-dwelling population.

## 2. Methods

### 2.1. Study Population

The present analysis consisted of 4587 Han Chinese at least 18 years of age and from a routine health check-up in Beijing between May 2007 and July 2009. A stratified cluster-sampling method was used. In the first stage of sampling, three districts (Fengtai, Shijingshan, and Daxing) were selected from 18 districts in Beijing; in the second stage of sampling, four communities were selected from these districts; and in the third stage of sampling, participants were selected from these communities. Elevated levels of serum ALT were defined as an enzyme activity > 40 U/L [13] and observed in 422 participants (9%). After these participants were excluded, there were 4165 participants in the final analysis.

### 2.2. Data Collection and Blood Sampling

Participants were asked to fast overnight (≥12 h) and attended the local health service center for their scheduled appointments. An interview using a structured questionnaire was conducted at the time of participants' visit. This questionnaire included the demographic (age and sex) and behavior (alcohol drinking). Height and weight were measured by well-trained physicians using the standard methods, and body mass index (BMI) was computed from weight in kilograms divided by height in meters squared. Participants were asked to remove two shoes and wear light clothes. Blood pressure was measured using a calibrated mercury sphygmomanometer (Yuwell Medical Equipment & Supply Co. Ltd., Jiangsu, China) after participants had been in the sitting position for ≥5 minutes. Blood pressure was measured two times consecutively, with ≥1 minute between measurements. Mean values of systolic and diastolic blood pressures (SBP and DBP) were used for statistical analysis. Blood sample was available for biochemical analysis and tested by qualified personnel blind to other information in our central laboratory. Serum levels of fasting blood glucose (FBG), triglyceride, low-density lipoprotein-cholesterol (LDL-c), high-density lipoprotein-cholesterol (HDL-c), and ALT were evaluated using the enzymatic assays (Roche Products Ltd., Basel, Switzerland) on a full automatic biochemical autoanalyzer (COBAS c6000; Roche Products Ltd., Basel, Switzerland). Alcohol drinking was defined as the consumption of at least 30 grams of alcohol per week for ≥1 year [14]. Hypertension was diagnosed by the presence of high blood pressure (≥140/90 mmHg) or using the antihypertensive medications. Diabetes mellitus (DM) was defined as elevated FBG levels (≥7.0 mmol/L) or using the antidiabetic medications.

### 2.3. Artery Pulse Wave Analysis

Pulse wave analysis was performed in the morning, in a quiet environment, and in the supine position to assess the AIx (HEM-9000AI; Omron Healthcare Inc., Kyoto, Japan). Radial artery was gently compressed with the tip of a tonometer at the site of maximal pulsation. The tonometer contains a micromanometer which provided a very accurate recording of the pressure within the artery. The system software was used to calculate an average radial artery waveform, and the corresponding central artery pressure waveform was generated using a transfer function of the instrument. AIx was defined as the ratio of augmentation to pulse pressure and expressed as a percentage. As there is a linear relationship between heart rate and AIx, AIx was standardized to a steady heart rate of 75 beats/minute. Resting heart rate was measured by cardiac auscultation for 1 minute.

### 2.4. Statistical Analysis

Continuous variables with normal distribution were expressed as mean and standard deviation. Continuous variables with skewed distribution were expressed as median and interquartile range. Categorical variables were expressed as number and percentage. Baseline characteristics were separated according to the quartiles of serum ALT levels as follows: quartile 1 (≤10 U/L), quartile 2 (11–20 U/L), quartile 3 (21–30 U/L), and quartile 4 (31–40 U/L) and compared using one-way analysis of variance for continuous variables with normal distribution, Kruskal-Wallis analysis for continuous variables with skewed distribution, and *x*^2^ analysis for categorical variables. To investigate the associations of serum ALT levels with cardiometabolic risk factors, the Pearson or Spearman correlation was used for univariate analysis, and multivariate linear regression analysis was performed after adjusting for age, sex, BMI, hypertension, DM, SBP, DBP, HR, triglyceride, HDL-c, LDL-c, FBG, and AIx. Drinking dosage was used as an additional adjustment factor in multivariate analysis of drinkers. Serum ALT levels were logarithmically transformed for a better fit of multivariate linear regression analysis. Statistical analysis was performed separately for nondrinkers and drinkers using the Statistical Package for Social Sciences (SPSS) version 17.0 (SPSS Inc., Chicago, IL, USA), with a two-tailed *p* value less than 0.05 set as significant.

## 3. Results

Median (range) age of the total population was 51 (18–96) years, and 46.9% (1953 participants) were men. Alcohol drinking was observed in 1173 participants (28.2%). Drinking dosage had a median (range) of 117 (31–1167) grams per week.  Table 1 shows the characteristics of nondrinkers according to the quartiles of serum ALT levels. Participants between groups had significantly different levels of age, BMI, SBP, DBP, triglyceride, HDL-c, LDL-c, FBG, and AIx and proportions of sex, hypertension, and DM (*p* < 0.05 for all).  Table 2 shows the characteristics of drinkers according to the quartiles of serum ALT levels. Participants between groups had significantly different levels of BMI, SBP, DBP, HR, triglyceride, HDL-c, LDL-c, FBG, and drinking dosage and proportions of sex and DM (*p* < 0.05 for all).

Associations of serum ALT levels with cardiometabolic risk factors were evaluated firstly by univariate analysis and then by multivariate analysis (Table 3). In univariate analysis, serum ALT levels were significantly related to age, sex, BMI, hypertension, DM, SBP, DBP, triglyceride, HDL-c, LDL-c, FBG, and AIx in nondrinkers, while serum ALT levels were significantly related to sex, BMI, DM, SBP, DBP, triglyceride, HDL-c, LDL-c, FBG, and drinking dosage in drinkers (*p* < 0.05 for all). In multivariate analysis, serum ALT levels of nondrinkers were independently associated with age, sex, BMI, hypertension, DM, DBP, triglyceride, LDL-c, and AIx, while serum ALT levels of drinkers were independently associated with age, sex, BMI, triglyceride, and LDL-c (*p* < 0.05 for all). Serum ALT levels of drinkers were also independently associated with drinking dosage (*p* < 0.001).

## 4. Discussion

Cardiometabolic risk factors are increasingly identified, and patients with cardiometabolic risk factors often have elevated levels of serum ALT. Serum ALT levels not only are widely used to monitor the hepatic function in these patients [1] but also have been reported to be correlated with cardiometabolic risk factors, such as age, sex, body height and weight, and blood pressure, glucose, and lipid [2–5]. But these associations are not full analyzed within normal range of serum ALT levels, and thus, the significance of serum ALT levels within normal range needs to be evaluated in relation to cardiometabolic risk factors [6]. Additionally, the studies in clinical patients cannot reflect the associations between serum ALT levels and cardiometabolic risk factors in community-dwelling population, and these associations have been shown to vary in different ethnicities [7–9]. However, most studies about these associations have not been performed in Chinese community-dwelling population. The present analysis revealed that in drinkers, serum ALT levels within normal range were independently associated with age, sex, BMI, and blood pressure, glucose, and lipid, while in nondrinkers, serum ALT levels within normal range were only associated with age, sex, BMI, and blood lipid but not blood pressure and glucose, supporting the notion that the associations between serum ALT levels and cardiometabolic risk factors existed even within normal range of serum ALT levels. Meanwhile, alcohol drinking has direct effect on serum ALT levels, and the present analysis indicated that there were independent associations of serum ALT levels with blood pressure and glucose in nondrinkers rather than drinkers [15].

In addition to traditional cardiometabolic risk factors, AIx is a useful tool to evaluate the arterial compliance, and its decline is paralleled with increased arteriosclerosis [10–12]. However, to our knowledge, the association between serum ALT levels and AIx has not been documented all around the world, and this is the first study to investigate this association in Chinese community-dwelling population. Moreover, AIx is significantly affected by age, sex, body height and weight, and blood pressure, glucose, and lipid, and multivariate analysis was performed after adjusting for these traditional cardiometabolic risk factors in the present analysis. The present analysis found that in nondrinkers rather than in drinkers, there was an independent association between serum ALT levels and AIx even within the normal range of serum ALT levels, suggesting not only the existence of association between hepatic function and arteriosclerosis risk but also the effect of alcohol drinking on this association. In previous studies, serum ALT levels are the most suitable biomarker of nonalcoholic steatohepatitis as the hepatic enzyme most closely correlated with hepatic lipid accumulation [16]. In hepatic disease, elevated levels of serum ALT are more significantly associated with nonalcoholic steatohepatitis than with other causes of hepatitis (alcoholic hepatitis, chronic hepatitis B and C, and autoimmune hepatitis) [15], and it is no wonder that nonalcoholic steatohepatitis has more significant associations with arteriosclerosis and cardiometabolic risk factors than other types of hepatitis.

In conclusion, this large Chinese community-based analysis demonstrated that the associations of serum ALT levels within normal range with age, sex, body height and weight, and blood lipid were simultaneously present in participants with and without alcohol drinking, while the associations of serum ALT levels within normal range with AIx, blood pressure, and glucose were seen in nondrinkers rather than in drinkers. These findings not only provide the evidence that serum ALT levels, even within the normal range, have different associations with arteriosclerosis and cardiometabolic risk factors in nondrinkers and drinkers but also are helpful in understanding the underlying pathophysiologic mechanisms linking the hepatic function to arteriosclerosis and cardiometabolic risk factors.

## Figures and Tables

**Table 1:** Characteristics of nondrinkers according to the quartiles of serum ALT levels.

Characteristics	Quartile 1 ≤10 U/L (*n* = 347)	Quartile 2 11–20 U/L (*n* = 1656)	Quartile 3 21–30 U/L (*n* = 727)	Quartile 4 31–40 U/L (*n* = 262)	*p* value
Age (year)	44 (33–59)	54 (43–67)	54 (45–63)	52 (44–59)	<0.001
Men (%)	51 (14.7)	436 (26.3)	279 (38.4)	115 (43.9)	<0.001
BMI (kg/m^2^)	22.97 (20.70–25.76)	24.98 (22.76–27.70)	26.49 (24.04–28.91)	26.95 (24.98–29.68)	<0.001
Hypertension (%)	77 (22.2)	649 (39.2)	362 (49.8)	127 (48.5)	<0.001
DM (%)	18 (5.2)	176 (10.6)	94 (12.9)	41 (15.6)	<0.001
SBP (mmHg)	116 (106–129)	124 (113–139)	129 (119–142)	127 (118–142)	<0.001
DBP (mmHg)	72 (68–80)	77 (70–81)	80 (71–86)	80 (72–87)	<0.001
HR (beats/minute)	76 (70–83)	75 (69–82)	75 (68–82)	75 (69–83)	0.095
Triglyceride (mmol/L)	0.97 (0.75–1.33)	1.27 (0.90–1.74)	1.52 (1.06–2.19)	1.62 (1.16–2.41)	<0.001
HDL-c (mmol/L)	1.54 (1.30–1.79)	1.43 (1.22–1.69)	1.34 (1.15–1.58)	1.31 (1.13–1.52)	<0.001
LDL-c (mmol/L)	2.41 (1.95–2.95)	2.75 (2.28–3.27)	2.91 (2.46–3.38)	3.03 (2.57–3.43)	<0.001
FBG (mmol/L)	4.86 (4.57–5.16)	4.95 (4.60–5.38)	5.05 (4.67–5.45)	5.15 (4.78–5.60)	<0.001
AIx (%)	79 (70–88)	82 (74–89)	82 (74–89)	82 (72–91)	0.015

ALT: alanine aminotransferase; BMI: body mass index; DM: diabetes mellitus; SBP: systolic blood pressure; DBP: diastolic blood pressure; HR: heart rate; HDL-c: high-density lipoprotein-cholesterol; LDL-c: low-density lipoprotein-cholesterol; FBG: fasting blood glucose; AIx: augmentation index.

**Table 2:** Characteristics of drinkers according to the quartiles of serum ALT levels.

Characteristics	Quartile 1 ≤10 U/L (*n* = 92)	Quartile 2 11–20 U/L (*n* = 595)	Quartile 3 21–30 U/L (*n* = 339)	Quartile 4 31–40 U/L (*n* = 147)	*p* value
Age (year)	44 (31–63)	46 (34–58)	45 (34–55)	43 (35–53)	0.109
Men (%)	71 (77.2)	540 (90.8)	321 (94.7)	140 (95.2)	<0.001
BMI (kg/m^2^)	23.57 (21.41–25.79)	24.87 (22.95–26.85)	25.85 (23.85–27.72)	27.04 (25.46–29.00)	<0.001
Hypertension (%)	21 (22.8)	192 (32.3)	109 (32.2)	52 (35.4)	0.226
DM (%)	4 (4.3)	34 (5.7)	20 (5.9)	18 (12.2)	0.023
SBP (mmHg)	120 (113–130)	125 (117–137)	126 (116–136)	126 (118–138)	0.017
DBP (mmHg)	74 (68–80)	78 (71–83)	80 (72–87)	80 (72–88)	<0.001
HR (beats/minute)	74 (67–82)	72 (66–79)	74 (66–82)	74 (68–82)	0.049
Triglyceride (mmol/L)	0.92 (0.73–1.26)	1.25 (0.89–1.72)	1.54 (1.09–2.23)	1.72 (1.24–2.77)	<0.001
HDL-c (mmol/L)	1.53 (1.35–1.80)	1.39 (1.19–1.59)	1.32 (1.14–1.54)	1.26 (1.03–1.50)	<0.001
LDL-c (mmol/L)	2.20 (1.85–2.80)	2.54 (2.12–3.02)	2.68 (2.27–3.10)	2.76 (2.29–3.35)	<0.001
FBG (mmol/L)	4.87 (4.53–5.20)	4.89 (4.59–5.19)	4.98 (4.66–5.33)	5.01 (4.68–5.52)	0.002
AIx (%)	70 (60–80)	72 (62–83)	71 (60–81)	72 (61–82)	0.470
Drinking dosage	42 (34–57)	95 (54–140)	167 (80–279)	257 (117–500)	<0.001

ALT: alanine aminotransferase; BMI: body mass index; DM: diabetes mellitus; SBP: systolic blood pressure; DBP: diastolic blood pressure; HR: heart rate; HDL-c: high-density lipoprotein-cholesterol; LDL-c: low-density lipoprotein-cholesterol; FBG: fasting blood glucose; AIx: augmentation index.

**Table 3:** Associations of serum ALT levels with cardiometabolic risk factors in univariate and multivariate analyses.

Variables	Nondrinkers				Drinkers					
	Univariate		Multivariate		Univariate		Multivariate		Multivariate	
	*r*	*p* value	*β*	*p* value	*r*	*p* value	*β*	*p* value	*β*	*p* value
Age (year)	0.082	<0.001	−0.117	<0.001	−0.055	0.059	−0.162	<0.001	−0.126	<0.001
Men/women	0.199	<0.001	0.234	<0.001	0.167	<0.001	0.162	<0.001	0.110	<0.001
BMI (kg/m^2^)	0.302	<0.001	0.208	<0.001	0.303	<0.001	0.229	<0.001	0.191	<0.001
Hypertension (%)	0.177	<0.001	0.054	0.024	0.057	0.050	−0.007	0.866	<0.001	0.997
DM (%)	0.094	<0.001	0.061	0.004	0.063	0.032	0.052	0.118	0.030	0.322
SBP (mmHg)	0.205	<0.001	−0.044	0.114	0.082	0.005	−0.035	0.422	−0.060	0.129
DBP (mmHg)	0.230	<0.001	0.088	<0.001	0.167	<0.001	0.078	0.055	0.082	0.051
HR (beats/minute)	−0.022	0.229	−0.002	0.893	0.035	0.225	0.002	0.949	−0.014	0.572
Triglyceride (mmol/L)	0.299	<0.001	0.150	<0.001	0.331	<0.001	0.197	<0.001	0.176	<0.001
HDL-c (mmol/L)	−0.183	<0.001	0.013	0.517	−0.195	<0.001	0.012	0.688	−0.008	0.767
LDL-c (mmol/L)	0.206	<0.001	0.134	<0.001	0.159	<0.001	0.086	0.003	0.076	0.004
FBG (mmol/L)	0.136	<0.001	−0.021	0.320	0.124	<0.001	0.028	0.412	0.049	0.110
AIx (%)	0.036	0.049	0.073	<0.001	−0.004	0.882	0.062	0.074	0.042	0.179
Drinking dosage	—	—	—	—	0.443	<0.001	—	—	0.375	<0.001

ALT: alanine aminotransferase; BMI: body mass index; DM: diabetes mellitus; SBP: systolic blood pressure; DBP: diastolic blood pressure; HR: heart rate; HDL-c: high-density lipoprotein-cholesterol; LDL-c: low-density lipoprotein-cholesterol; FBG: fasting blood glucose; AIx: augmentation index.
